# Characteristics of Patients Referred To A Specialized Headache Clinic

**DOI:** 10.1038/s41598-020-58234-w

**Published:** 2020-01-24

**Authors:** Eva Fejes, Gergely Feher, Zsuzsanna Gurdan, Katalin Gombos, Katalin Koltai, Gabriella Pusch, Antal Tibold

**Affiliations:** 1 Centre for Occupational Medicine, Medical School, University of Pécs, Pécs, Hungary; 2 Hospital of Komlo, Komlo, Hungary; 3 Neurology Ward, Hospital of Szigetvar, Szigetvar, Hungary; 4 Department of Paediatric and Adolescent Dentistry, Medical School, University of Pécs, Pécs, Hungary; 5 Department of Laboratory Medicine, Medical School, University of Pécs, Pécs, Hungary; 6 First Department of Medicine, Medical School, University of Pecs, Pécs, Hungary; 7 Department of Neurology, Medical School, University of Pecs, Pécs, Hungary

**Keywords:** Epidemiology, Migraine

## Abstract

Headache is a common problem with great effect both on the individual and on the society. Recent studies raised the possibility of increasing rate of specialty referrals, inappropiate treatment and advanced imaging for simple headache. The aim of our study was to analyze the characteritics of patients (including duration of symptoms, headache type, brain imaging, treatment) referred to our specialized headache clinic between 01/01/2014 and 01/01/2015 by their general practitioners and primary care neurologists due to chronic/treatment-resistant headache syndromes. 202 patients (mean age 53.6 ± 17.6 years) were evaluated in our clinic (102 females, mean age 50.14 ± 16.11 years and 100 males, mean age 57 ± 18.1 years). Migraine (84/202) and tension-type (76/202) were the most common syndromes. 202 plain brain CT, 60 contrast-enhanced CT and 128 MRI were carried out by their general practitioners or other healthcare professioners including neurologists before referral to our headache centre. Despite of extensive brain imaging appropiate treatment was started less than 1/3 of all patients and significant proportion received benzodiazepines or opioid therapy. Furthermore, more than 10% of referred patients presented with secondary headache including one meningitis. The management of headache is still a challenge for primary care physicians leading to medical overuse. Vast majority of our patients should not be referred to our specialized headache clinic as they had uncomplicated headache or other underlying conditions than pain.

## Introduction

Headache is a common problem with great effect both on the individual and on the society^1^. The rate of migraineurs is approximately 10% in the whole population and it is highly possible that 30–40% suffers from tension-type headache^2^. These headaches (and most primary headaches) are episodic but can transform into chronic form. Symptoms can be frightening for the patients including severe pain, nausea, vomiting. Its consequences include inability to work or reduced productivity and can provoke anxiety, avoidance behaviour, unnecessary hospital admissions and brain imaging^1,3^.

As headache disorders are amongst the leading cause of years lived with disability worldwide (migraine alone is ranked as third among people aged 15 to 49 years) to improve the management of patients with headache, the Hungarian Headache Society established 29 Specialized Headache Centers, which accept referrals from general practitioners (and other medical professioners) or from neurologists not specialized in headache^4,5^. Our specialized headache center was established in 2014 in Szigetvar, accepting referrals from 3 primary hospitals, 4 general outpatient clinics and 25 general practitioners, overall covering more than 70000 patients in South West Hungary^4^.

Recent studies showed the increasing rate of specialty referrals and advanced imaging for simple headache which can result in unnecessary hospital admissions^1,6^. Patients may have to take a long journey until getting to a specialist to receive appropiate treatment^1,6^.

Furthermore, there is a link between primary headaches and stroke with gender-dependent, age-specific and time-dependent characteristics^7^. However, there is no evidence focusing on preventive treatment, but careful evaluation of cardiovascular risk factors is reasonable^8^.

As only several reports (and no studies from our country) are available with regard to primary care management of headache patients we overtook a retrospetive study to analyze the characteritics of patients (including duration of symptoms, headache type, brain imaging, treatment and cardiovascular risk factors) referred to our headache clinic by their general practitioners and primary care neurologists due to chronic/treatment-resistant headache syndromes.

## Patients and Methods

202 patients were referred to our outpatient service between 01/01/2014 and 01/01/2015 and data were retrospectively analyzed.

Headache type was classified due to the International Headache Society (IHS) guidelines^9^. Duration of symptoms, brain imaging (including plain computer tomograph /CT/, contrast-enhanced CT and magnetic resonance imaging /MRI/), previous outpatient/hospital attendance due to headache and treatment strategies were extracted from hospital notes.

Cardiovascular risk profile factors and previous diseases of relevance to this study included, smoking habit, diabetes mellitus, hypertension, dyslipidaemia, ischaemic heart disease (IHD), history of stroke and peripheral artery disease.

A concomitant medication history was taken with respect to use of beta-adrenoreceptor blockers, angiotensine converting enzyme (ACE) inhibitors, angiotensine (AT) II receptor blockers and statins.

Data were evaluated as means ± SD (standard deviation) by Student’s t-test and the chi square test.

### Ethics approval and consent to participate

The study protocol conforms to the ethical guidelines of the 1975 Declaration of Helsinki as reflected in a priori approval by the Regional Ethical Committee at the Faculty of Medicine, University of Pécs. Informed consent were not given to the patients due to the retrospective nature of the study.

## Results

Between 01/01/2014 and 01/01/2015, 202 patients (mean age 53.6 ± 17.6 years) were evaluated in our clinic (102 females, mean age 50.14 ± 16.11 years and 100 males, mean age 57 ± 18.1 years). Males were significantly older than females (p < 0.01). Duration of symptoms were 9.3 years overall (11.6 years in females and 7.1 years in males, p < 0.01). 202 plain brain CT (94 for females and 108 for males), 60 contrast-enhanced CT (30 for males and 30 for females) and 128 MRI examinations (68 for females and 60 for males) were carried out due to exclude other etiology than headache by their general practitioners (GPs) or other healthcare professioners including neurologists before referral (Table 1).

**Table 1:** Baseline data, brain imaging and emergency observations in the study populations.

		Patient number	Mean age (years)	Duration of symtoms (years)	Brain CT (number)	Contrast-enhanced brain CT (number)	Brain MRI (number)
Study population		202	53.6 ± 17.6	9.3	202	60	128
	Males	100	57 ± 18.1	7.1	108	30	60
	Females	102	50.14 ± 16.11*	11.6*	94	30	68
Migraine		84	46.1 ± 14.7	13.8	68	32	66
	Males	18	42.22 ± 11.3	12.2	10	2	14
	Females	66	47.12 ± 15.3	14.2	58	30*	52
Tension-type headache		76	59.66 ± 17.9	6.81	100	16	46
	Males	54	54.18 ± 18.4	6.74	74	12	36
	Females	22	61.8 ± 17.3*	7	26	4	10
Trigeminal-autonomic cephalalgia		18	45.33 ± 12.3	6.16	14	14	12
	Males	8	35.4 ± 5.1	7.3	6	7	5
	Females	10	57.7 ± 5.1*	4.75	8	7	7
Secondary headaches		24	67 ± 11.82	2.62	18	0	4
	Males	20	69 ± 10.68	2.4	14	0	3
	Females	4	61 ± 14*	4	4	0	1

*p < 0.05 among females and males within the subgroups.

Migraine was diagnosed in 84 patients (mean age 46.1 ± 14.7 years) corresponding to the IHS criteria (66 females, mean age 47.12 ± 15.3 years and 18 males, mean age 42.22 ± 11.3 years). All patients fulfilled the IHS criteria of chronic migraine^9^. Mean age was not significantly different among the genders (p = 0.1). 20 patients had migraine with aura (14 with visual aura, 4 with sensory and 2 with motoric aura) and 6 patients had vestibular migraine. Duration of symptoms were 13.8 years overall (14.2 years for females and 12.2 years for males, p = 0.21) (Table 1).

Overall 68 plain brain CT (58 for females and 10 for males, p = 0.28), 32 contrast-enhanced CT (30 for females and 2 for males, p < 0.05) and 66 MRI examinations (52 for females and 14 for males, p = 0.89) were carried out. Patients had an average of 5.2 emergency observations/admissions (5.9 for females and 2.4 for males p < 0.05) (Table 1). As all patients had at least one brain imaging before referral, no further imaging modalities were required to arrange by us due to long standing pain and the absence of red flags.

Non steroidal anti-inflammatory drugs (NSAIDs) (without any preference) were prescibed for all patients and triptans were prescibed for 16% (14/84). 25% of patients (21/84) received prophylactic treatment in accordance with the current European guidelines^10^. Selective serotonin reuptake inhibitors (SSRIs) were prescibed for 6 patients (7%) and benzodiazepines (BDZs) for 8 patients (9.5%) by their GPs without any clinical evidence of depression or anxiety. Opoioids for pain relief were prescribed for 16 patients (19%) (Table 2).

**Table 2:** Treatment strategies in primary headache syndromes.

	Migraine (number)	Tension-type headache (number)	Trigeminal-autonomic cephalagia(number)
Triptanes	14/84	0/76	0/18
Prophylactic treatment	21/84	22/76	0/18
Opioids	16/84	10/76	5/18
BDZs	8/84	17/76	5/18
SSRIs)	6/84	8/76	0/18

Abbreviations: benzodiazepines (BDZs), selective serotonin reuptake inhibitors (SSRIs).

Tension-type headache was diagnosed in 76 patients (mean age 59.66 ± 17.9 years) corresponding to the IHS criteria (22 females, mean age 61.8 ± 17.3 years and 54 males, mean age 54.18 ± 18.4 years, p < 0.05). All patients fulfilled the IHS criteria of chronic tension-type headache (headache occurring on ≥15 days/month on average for >3 months)^11^. Duration of symptoms were 6.81 years overall (7 years for females and 6.74 years for males, p = 0.43) (Table 1). Moreover, patients with tension-type headache were significantly older than migraineurs and had more vascular co-morbidities (p < 0.05) (Table 3).

**Table 3:** Cardiovascular risk factors and treatment strategies in primary headache syndromes.

	Migraine (%)	Tension-type headache (%)
Smoking	37	33
Hypertension	57	80*
Diabetes	8	33.3*
Peripheral arterial disease	0	0
TIA/stroke	0	11.1*
IHD	6	38.8*
ACE inhibitor	34	55.5*
ARB	14	16
Ca-chanel inhibitor	11	36*
Beta-blocker	25	47*
Statin	8	33*

*p < 0.05 between migraneurs and patients with tension-type headache.

Abbreviations: transient ischemic attack (TIA), ischemic heart disease (IHD), angiotensin-converting-enzyme inhibitor (ACE inhibitor*), a*ngiotensin II receptor blockers (ARB), calcium chanel (Ca-chanel).

Overall 100 plain brain CT (26 for females and 74 for males, p = 0.66), 16 contrast-enhanced CT (4 for females and 12 for males, p = 0.74) and 46 MRI examinations (10 for females and 36 for males, p = 0.76) were carried out. Patients had an average of 5.6 emergency observations/admissions (4 for females and 6.3 for males p = 0.07) (Table 1). As all patients had at least one brain imaging before referral, no further imaging modalities were required to arrange by us due to long standing problems and the absence of red flags.

NSAIDs (without any preference) were prescribed for all patients and triptans were not prescibed at all. 29% (22/76) of patients received treatment in accordance with the current European guidelines^9^. SSRIs were prescibed for 8 patients (10.5%) and BDZs for 17 patients (22.4%) by their GPs without any clinical evidence of depression or anxiety. Opoioids for pain relief were prescribed for 10 patients (13.1%) (Table 2).

Trigeminal-autonomic cephalagia was diagnosed in 18 patients (mean age 45.33 ± 12.3 years) corresponding to the IHS criteria (8 females, mean age 57.7 ± 5.1 years and 10 males, mean age 35.4 ± 5.1 years, p < 0.05), including 15 cluster headaches, 1 short-lasting unilateral neuralgiform headache with conjunctival injection and tearing (SUNCT) syndrome and 2 hemicrania continua headache^12^. Duration of symptoms were 6.16 years overall (4.75 years for females and 7.3 years for males) (Table 1).

Overall 14 plain brain CT (8 for females and 6 for males), 14 contrast-enhanced CT (7 for females and 7 for males) and 12 MRI examinations (7 for females and 5 for males) were carried out before referral. Patients had an average of 5.8 emergency observations/admissions (6 for females and 5.6 for males) (Table 1). Due to the low patient number, gender differences were not calculated. Patients with trigeminal-autonomic cephalalgia had no significant cardiovascular risk factors apart from smoking (5%). Despite of previous brain imaging, to attach to the guidelines and to exclude other etiology than primary headache, several MRI scans were arranged by us further on (3 for cluster headaches, 1 for short-lasting unilateral neuralgiform headache with conjunctival injection and tearing (SUNCT) syndrome and 2 for hemicrania continua headache) as they had only a plain CT before referral. Triptans, indomethacin and steroids were not prescibed at all. No patients received treatment in accordance with the current European guidelines^12^. Opoioids and BDZs for pain relief were prescribed for 5-5 patients (25-25% (Table 2).

24 patients had secondary headache or other etiology than headache (not shown). One patient had subacute meningitis, 2 patients had atypical facial pain, 2 patients had subacute stroke with headache, 2 patients with previously known non-Hodgkin lymphoma had brain propagation/metastasis, 6 patients had peripheral vertigo and 12 patients had psychiatric comorbidity with secondary headache.

All patients with psychiatric comorbidity and peripheral vertigo had plain CT or MRI scans, but no contrast-enhanced CTs were carried out (not shown). We have arranged CT scans for those presenting with subacute stroke and MRI scans for those presenting with space occupying lesions.

NSAIDs were prescibed for all patients, and BDZs were used for 12/24 (50%) patients and opiodis were prescibed for 8/24 (33%) patients.

## Discussion

Headache is a common problem, burdening both the individual and the society. Based on our results about 20% of headache patients were properly managed in primary care which resulted in unnecessary emergency admissions and enourmous number of brain imaging.

Although headache symptoms can be frightening for the patients there is a strong recommendation against routine scanning of headache patients. Surprisingly, despite of strong recommendation against routine scanning of headache patients our patients were scanned usually more than once for an uncomplicated headache by their GPs or primary care physicians including neurologists. The diagnostic yield of plain CT and contrast-enhanced CT scans (apart from emergency situations such as probable subarachnoideal hemorrhage or brain injury) is very limited in the diagnotic work-up of headache^13,14^. Furthermore, ionizing radiation can cause damage to deoxyribonucleic acid (DNA), increasing the risk of malignancies, especially in the case of recurrent scannings or dose exceedings^15–17^. About 4000 future cancers can be related to head scannings in the US and probably 2 percent of future cancers will be caused by radiation from unnecessary CT scannings^15–17^. Therefore the current European guidelines recommend MRI of the brain in primary headaches if necessary, but it merits in special circumstances only^13^. The unnecessary scanning of headache patients is not a unique finding in our region. In general, about one-third of all CT scans can be unnecessary and a constant increase of neuroimaging ordered during outpatient headache visits can be detected based on heathcare database systems in the US^15,17–20^.

Apart from NSAIDs, triptanes are the first line treatment of acute migraine, and they seem to be effective in tension-type headache^21^. However, these agent were prescibed for 10 percent of migraneurs and were not used in tension-type or trigeminal-autonomics cephalagias in our region.

Despite all guidelines recommend against opioids as first-line treatment for acute migraine and other primary headaches, they were prescribed for about 20% of our patients (with the highest rate in secondary headaches). This is in contrast with previous studies. A retrospective analysis of health system data showed that the rate of prescriptions of opioids/barbiturates can be as high as 16% for headache syndromes^22^.

However, opioids may be considered as adjunctive therapy for short-term relief in therapy-resistant cases, but their long term use can be associated with severe side effects, increased tolerancy and dependency^23,24^. Futhermore, the combination of different analgesics and opioids can precipitate the development of medication-overuse headache^24^.

BDZs were also frequently used. Albeit analgesic effects of classical BDZs have occasionally been reported, they are not recommended for migraine (and any pain) treatment^25,26^. Long-term intake can be associated with a significant increase of migraine occurrence^27^. SSRIs were also used for pain relief, but their efficacy in any primary headache in not supported by robust evidence^28^.

We have no obvious explanation to the relatively high prescription of these drugs. Vast majority of our patients fulfilled the criteria of chronic headache syndromes (those with migraine and tension-type headache) and were not properly managed in the primary care. Chronic pain (and headache) usually have neuropsyhiatric complications including mood disorders and insomnia which are usually treated with BDZs and SSRIs^29^.

Vast majority of our primary headache patients fulfilled the criteria of chronic headache, but less than one-third of them received proper prohylactic or maintenance therapy in accordance with the current European and Hungarian recommendations. This is in concodance with recent studies, the diagnosis and management of migraine (and other primary headache syndromes - especially chronic forms) are still a challenge for primary care physicians^30^.

Due to inappropiate primary care management, our patients had several emergency admissions, which were the most common in patients with trigeminal-automic cephalagia, females with migraine and males with tension-type headache. They were discharged in the absence of any significant diseases laeding to increased anxiety and BDZ intake. Anxiety (and BDZ use) seem to be associated with exacerbation of headache intensity^31^.

We have also detected the relatively high rate of cardiovascular risk factors of patients with migraine and tension type headache (especially the rate of smoking, diabetes and hypertension). Pain physicians must be aware the cardiovascular aspects of migraine and a holictic approach is required including the strict control of modifiable cardiovascular risk factors instead of focusing only on pain relief, and should be kept in mind that the risk of stroke became gradually apparent by different following time intervals beyond 2 years after first diagnosis based on recent studies^7,8^.

About 10% of the referrals had secondary headache, inclunding one meningitis, which underlines the deficiency of primary care, which is not unique in our country^32^.

Furthermore, the is a huge overlap among migraine, anxiety and depression and clinicans should be taken masked/atypical depression also account^33^. This is a subtype of depression, in which somatic symptoms dominate the clinical picture disguiding the underlying psychiatric disorder^34^.

The explanation of the overuse of brain imaging and the undertreatment of headache in the primary care can be multifactorial. First of all there is a pression of urgent head imaging from the public due to frightening from a severe disease^35^. There is also a low threshold to scan patients presenting with severe headaches in the emergency departments. Secondary, a significant proportion of junior doctors did not receive formal teaching on how to take a complete headache history and the vast majority of them have not attended at all an outpatient headache clinic^36^.

Headache impacts both the individual and the society. Chronic headache is usually more disabling than episodic one and usually undertreated^37^. The economic cost of migraine can be as high as £835 million annually in the UK. Although we do not have proper data the estimated yearly direct cost (loss of working days) in Hungary is approximately 70 million Euro (the minimum salary is 400 Euro is our country)^38^.

Finally, our article has some limitations. First, it was retrospective study in nature. Secondly, this study was conducted in a single headache center and a referral bias was inherently present in our study, does not reflect normal age and gender distribution of headache syndroms, and patients with long standing and disabling headaches were referred as it was conducted at a specialty care center. Thirdly, we did not evaluate the burden of headache such as disability and impact of headache by using an instrument.

In conclusion, our article highlights the burdens of headache in our region. This is the first report from Hungary with regard to the primary management of headache based ont he data of a specialty clinic. The management of this contiditon is still a challenge for primary care physicians leading to medical overuse. Vast majority of our patients should not be referred to our Headache Clinic as they had uncomplicated headache or other underlying conditions than pain. We also noticed the underutilization of appropiate treatment and the overuse of BDZs and opioids.

## Conclusion

1. The management of headache is still a challenge for primary care physicians leading to medical overuse.
2. Due to inappropiate primary care management, our patients had several emergency admissions.
3. Vast majority of our patients should not be referred to our Headache Clinic as they had uncomplicated headache or other underlying conditions than pain.

## Data Availability

The dataset supporting the conclusions of this article is available on request to the corresponding author.
